# Functional connectivity of the human face network exhibits right hemispheric lateralization from infancy to adulthood

**DOI:** 10.1038/s41598-023-47581-z

**Published:** 2023-11-27

**Authors:** Keren Lesinger, Gideon Rosenthal, Karen Pierce, Eric Courchesne, Ilan Dinstein, Galia Avidan

**Affiliations:** 1 Department of Psychology, Ben-Gurion University of the Negev, POB 653, 8410501 Beer Sheva, Israel; 2 Department of Cognitive and Brain Sciences, Ben-Gurion University of the Negev, POB 653, 8410501 Beer Sheva, Israel; 3 Department of Neurosciences, University of California, San Diego, USA

**Keywords:** Perception, Human behaviour

## Abstract

Adults typically exhibit right hemispheric dominance in the processing of faces. In this cross-sectional study, we investigated age-dependent changes in face processing lateralization from infancy to adulthood (1–48 years old; N = 194). We co-registered anatomical and resting state functional Magnetic Resonance Imaging (fMRI) scans of toddlers, children, adolescents, and adults into a common space and examined functional connectivity across the face, as well as place, and object-selective regions identified in adults. As expected, functional connectivity between core face-selective regions was stronger in the right compared to the left hemisphere in adults. Most importantly, the same lateralization was evident in all other age groups (infants, children, adolescents) and appeared only in face-selective regions, and not in place or object-selective regions. These findings suggest that the physiological development of face-selective brain areas may differ from that of object and place-selective areas. Specifically, the functional connectivity of the core-face selective regions exhibits rightward lateralization from infancy, years before these areas develop mature face-selective responses.

## Introduction

Faces have a distinctive evolutionary and social significance for humans. It is, therefore, not surprising that face perception is probably the most developed visual perceptual skill in humans. Consistent with this account is evidence implying innate, genetic^1–6^ as well as environmental components in face perception^7–9^.

In adults, a distributed cortical and subcortical network has been implicated in face perception^10^. This network exhibits right hemispheric dominance, which is evident in stronger face-selective responses and stronger anatomical and functional connectivity^11–13^. Yet, little is known about how this lateralization develops during early life. Most studies that examined the development of the face network focused on changes in volume, face selectivity, and anatomical location of specific regions in the core network, particularly the Fusiform Face Area (FFA), and mostly were conducted with children aged 5 years and older due to methodological challenges associated with scanning younger children and babies^14–19^. Yet, the characterization of the exact changes eliciting this pattern, as well as the corresponding ages in which various changes occur (e.g., volumetric changes, increase in face selectivity) are still a matter of ongoing debate. Several studies also revealed developmental changes that were unique to face-selective regions and were not evident in regions that corresponded to other visual categories^2,15,20,21^. For example, a quantitative Magnetic Resonance Imaging (qMRI) study with 5–12 years old children and adults revealed microstructural proliferation changes in face-selective regions but not in place-selective regions. These changes were also correlated with functional selectivity for faces and improved performance on face recognition tasks^20^. A number of studies have succeeded in showing face selectivity in young infants and even newborns using Event-Related Potentials (ERP)^22^ and fMRI. For example, in one study, 4 to 6 months old infants exhibited face selective responses in regions that were similar to those found in adults, but the magnitude of selectivity for faces was not as strong as in adults^15^. In contrast, a later study reported that infants between 2 to 9 months exhibited face selective responses in the FFA that were comparable in magnitude to those of adults^2^. This suggests that face selective responses are apparent in infancy, yet may still vary in their magnitude of selectivity.

While face selectivity appears early in life, the laterality of face selective brain regions seems to change throughout development, however the characteristics of its trajectory are still debated. Previous studies found that children exhibited similarly strong bilateral fMRI responses to faces, while adolescents exhibited rightward lateralized responses that were similar to those found in adults^23–25^. In addition, another study found that the right, but not left FFA showed an increase in volume as a function of age, suggesting a developmental change in lateralization of this area^17^. In contrast, ERP studies have implied that right lateralized face selective responses already appear in early infancy with lateralized P400 responses that are similar to the lateralized N170 responses found in adults^26^. There are, therefore, conflicting studies regarding how early face responses become right lateralized.

Beyond the cortical specialization of the core face areas described above, face processing is ultimately accomplished by the coordinated activity of multiple different regions including additional, more anterior areas of the extended face system, such as the anterior temporal cortex^27–31^. In that context, Johnson^32^ suggested the Interactive Specialization (IS) model, which implies that cortical specialization develops from the interaction between activity in different regions. This motivates the study of the face network in infants and toddlers using functional connectivity techniques. A recent study, revealed that early connectivity (at the age of 27 days) of the face-network was stronger to foveal than peripheral V1, in contrast to the connectivity of the place/scene network that was stronger to peripheral V1^21^. These results imply that early development of face-selective brain areas that later achieve face selectivity requires foveal input^7^. The other few existing studies, suggest an overall increase in functional connectivity within the whole face-network during childhood^33–35^. Specifically, a previous study found reorganization of the face network in children ages 7 to 13 years old which involved stronger functional connectivity between the core-face selective regions, with weaker functional connectivity (segregation) between the core and the extended face selective regions^35^. Yet, to the best of our knowledge, no study directly examined the emergence of the right hemispheric dominance during development using functional connectivity.

The goal of the current cross-sectional study was to characterize age-dependent changes in resting state functional connectivity between face-selective cortical areas from infancy to adulthood. Specifically, we focused on age-related functional connectivity changes in lateralization, while contrasting changes apparent in face versus object and place selective areas. We incorporated resting state fMRI data from several openly shared repositories, enabling us to assess a large number of participants 1–65 years old. To perform comparisons across age groups while addressing the significant anatomical brain changes that occur between infancy and adulthood^36^, we generated an average common brain template by morphing the brains of our participants^37^. This allowed us to define face, object, and place selective areas that were identified with a localizer scans conducted with adults^30^. We then examined functional connectivity in these areas and compared it across the different age groups.

## Methods

### Databases and participants

The final experimental groups included: adults with a localizer scan (N = 35) and 5 groups with resting state scans: (1) toddlers (N = 20, M = 2.52, sd = 0.90) (2) children (N = 34, M = 10.05, sd = 1.21), (3) early adolescents (N = 29, M = 13.56, sd = 0.85), late adolescents (N = 35, M = 16.56, sd = 0.90), (5) adults (N = 41, M = 25.78, sd = 7.97). (See details regarding the exclusion criteria below under “Preprocessing” and in the Supplementary Materials—Table S1—Site parameters and subject inclusion online).

Data from 3 different sources were included in the current study.

### Adult scans from Ben Gurion University (BGU)

Data was collected in Soroka University Medical Center, Beer Sheva, Israel^30^ using a 3 T Philips Ingenia scanner equipped with a standard head coil. fMRI Blood Oxygenation Level Dependent (BOLD) contrast was acquired using gradient-echo echo-planner imaging sequence. Specific scanning parameters were as follows: whole brain coverage 35 slices, transverse orientation, 3 mm thickness, no gap, TR = 2000 ms, TE = 35 ms, flip angle = 90°, FOV = 251 × 251 and matrix size 96 × 96. High-resolution anatomical volumes were acquired with a T1-weighted 3D pulse sequence (1 × 1 × 1 mm^3^, 170 slices) to allow coregistration of functional data. These data include 35 healthy adults with an anatomical scan, resting state fMRI scans and data from a face localizer. During the localizer scan, participants viewed blocks of faces and other stimuli such as places and objects and performed a one-back task^30^. 15 subjects had 2 localizer scans and 20 subjects had one localizer scan. The data was acquired as part of a study approved by the Helsinki committee of the Soroka University Medical Center, Beer Sheva, Israel. The study was performed in accordance with the relevant ethical guidelines and regulations. All subjects signed an informed consent form and received compensation for participating in the study.

### Children, adolescents, and adult scans from the Autism Brain Imaging Data Exchange (ABIDE)

Data included typically developed children, adolescents and adult participants 7–64 years old from the ABIDE database^38^. Data included anatomical and resting state fMRI scans. The data in the ABIDE database is fully anonymized as required by The Health Insurance Portability and Accountability Act (HIPAA) regulations and all participating sites received local Institutional Review Board approval for acquisition of the contributed data. The studies in each site were performed in accordance with the relevant ethical guidelines and regulations. In addition, informed consent was received from all the participants or from their parents in case they were under 18. The data, ethical requirements and its specific scanning parameters of each sample are available online at fcon_1000.projects.nitrc.org/indi/abide. (See detailed table with scanning parameters in Supplementary Materials—Table S1—Site parameters and subject inclusion online).

### Toddler scans from the University of California San Diego (UCSD)

Data included scans of typically developing toddlers previously studied by Dinstein et al.^39^. Anatomical and resting state fMRI scans were acquired in a GE 1.5 T Signa scanner located at the UCSD Radiology Imaging Laboratory in Sorrento Valley, CA. Scanning was performed with a standard GE birdcage head coil. fMRI BOLD contrast was obtained using a T2-sensitive echo planar imaging sequence (repetition time of 2000–2500 ms with 150–288 time points in length depending on the precise protocol used, 31 slices, 3 × 3 × 3 mm voxels). Anatomical volumes were acquired with a T1-weighted SPoiled Gradient-Recalled (SPGR) pulse sequence (0.94 × 0.94 × 1.2 mm). The study procedures for these data were approved by the UCSD human subject research protection program. The study was performed in accordance with the relevant ethical guidelines and regulations. All parents provided written informed consent and were paid for their participation. Specific details of the data collection used in this section can be found in Dinstein et al.^39^.

A total of 637 subjects were initially evaluated based on the following exclusion criteria (see details below under “Preprocessing” and in the Supplementary Materials—Table S1—Site parameters and subject inclusion online). Following anatomical and functional preprocessing, a subset of 194 participants, who passed the stringent criteria we applied, were included and a common space was created based on their anatomical template (see under “Subject normalization—creating a common space”). Details regarding resting state parameters varied across sites and datasets and are listed in Supplementary Materials—Table S2—Instructions given to participants in each resting state experiment in each site online.

### fMRI data analysis

Data analyses were performed using the fMRIB's FSL package (www.fmrib.ox.ac.uk/fsl). Common space was generated using the ANTs-Advanced Normalization Tools software^37^ that is built on the Insight ToolKit (ITK) framework. The ITK was also used for format synchronizing between FSL and ANTs. In addition, we used ITK-SNAP to label regions of interest^40^.

### Preprocessing

Preprocessing of resting state scans was performed using the Configurable Pipeline for the Analysis of Connectomes (C-PAC)^41^ and included: slice time correction, motion correction, skull stripping, nuisance regression of the first 5 PCs of the signal from obtained from white matter and Cerebrospinal fluid (CSF)^42^, 6 motion parameters and linear and quadratic trends, followed by temporal filtering between 0.1 and 0.01 Hz and global signal regression. Finally, we applied a scrubbing threshold of 0.2 mm frame-wise displacement (FD), which indexes the movement of the head from one volume to the next and removes 1 TR before and 2 TR after excessive movement^43^. Following this procedure, we excluded participants in whom the remaining duration of the scan was shorter than 5.5 min, we also excluded participants with low signal-to-noise ratio (SNR < 1).

All subjects from Rosenthal et al.^30^ (BGU dataset) where included in the anatomical template (see details below under “Subject normalization—creating a common space”) and the functional regions of interest (fROIS) analysis, but only subjects that passed the inclusion criteria were included in the statistical analysis. Hence, following all these procedures, 194 subjects were included in the generation of the anatomical template and 179 were included in the statistical analysis. Functional preprocessing of the localizer runs was applied using FSL and included autocorrelation of the time course and motion correction were regressed out and a low-pass filter was applied. All anatomical scans were registered to a cropped Montreal Neurological Institute (MNI) image, which results in shared orientation and minimization of zero voxels in all scans.

### Motion analysis

Using one-way ANOVA, we compared the mean FD between the different age groups and found no effect for age (F (4,154) = 1.450, p = 0.220 η^2^_p_ = 0.036). This result, together with a scrubbing threshold of 0.2 frame-wise displacement^43^, and the motion regression that was included in the preprocessing procedure, suggest that differences in in-scanner motion across different groups could not account for the results reported in this study.

### Subject normalization—creating a common space

**Figure 1:**
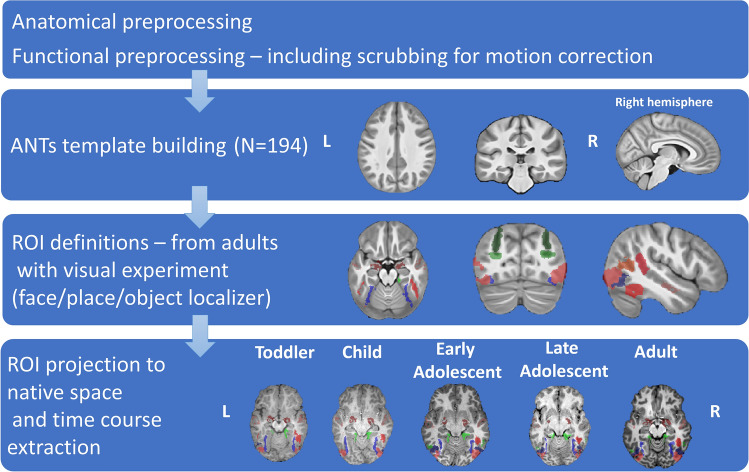
Workflow of preprocessing analysis. The template was based on participants aged 1–64 years old that passed the inclusion criteria. The projection of the fROIs is presented on brain scans of the participants scanned at BGU^30^, various sites from the ABIDE^38^ database and UCSD^39^ (see "Preprocessing" and Supplementary Materials—Table S1 online)*.* (*R* right hemisphere, *L* left hemisphere).

To address differences between infants and adults’ brains^44^, we used a unique method of “template building”, which is implemented in the ANTs (Advanced Normalization Tools) software (an extended description about the creation of the common space can be found in Avants et al.^37^ and Klein et al.^36^. This method is based on a symmetric diffeomorphic image normalization algorithm (SyN), an algorithm which is known for its capability to perform large deformation while preserving anatomical topography. We created a common standard space from different age groups in an iterative procedure which generated an average template of the anatomical scans (T1W) that shared the same orientation. All subjects’ scans were registered to this reference template using a non-linear registration and were then averaged. This average became the next reference template, and this procedure was iterated four times. The resulting template was considered as the common anatomical space, and it also contained the transformation matrices between each anatomical image and the template (see Fig. 1 under “Methods”).

### Functional ROIs analysis

**Figure 2:**
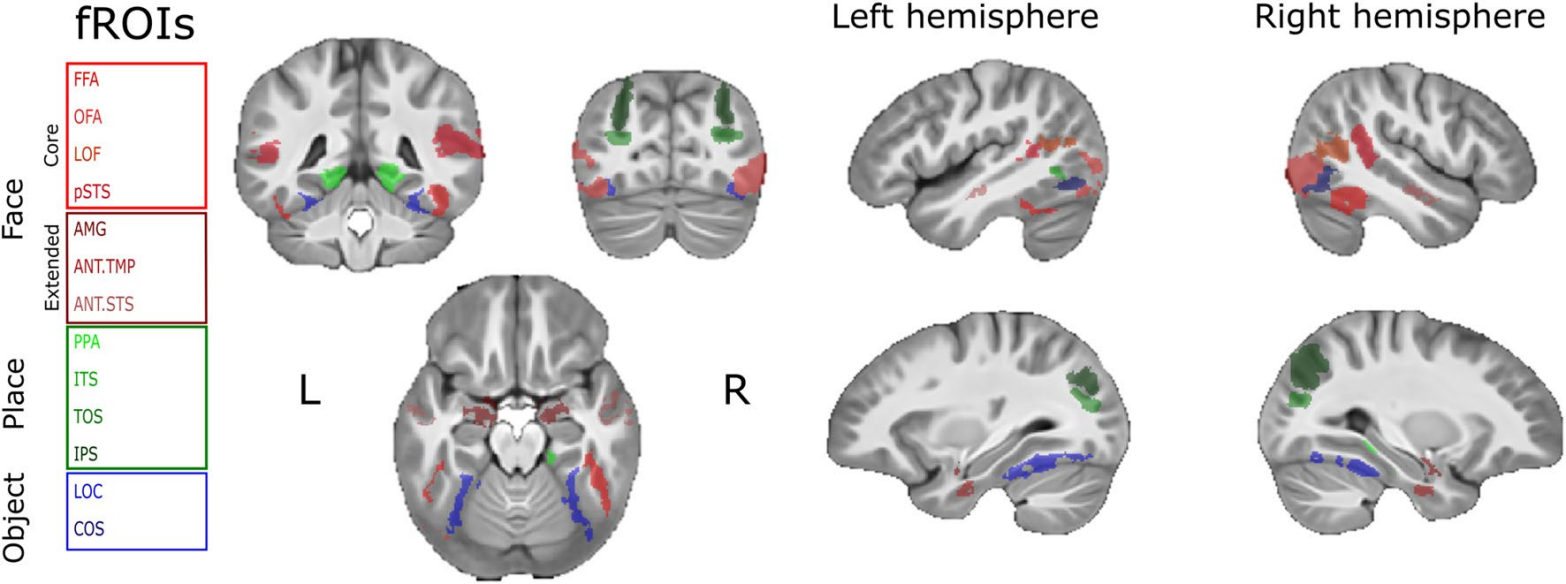
Functional ROIs of face, place and object selective regions projected on the resultant template space (see “Methods”—“Functional ROIs analysis”). (*R* right hemisphere; *L* left hemisphere; core-face selective regions: *FFA* fusiform face area, *OFA* occipital face area, *LOF* lateral occipital face area, *pSTS* posterior superior temporal sulcus; extended-face selective regions: *AMG* amygdala, *ANT.TMP* anterior temporal, *ANT.STS* anterior STS; place selective regions: *PPA* parahippocampal place area, *ITS* inferior temporal sulcus, *TOS* transverse occipital sulcus, *IPS* intraparietal sulcus; object selective regions: *LOC* lateral occipital cortex, *COS* collateral sulcus).

Resting state scans in children were taken from studies in which no face localizer was conducted. To define face, place, and object selective ROIs we used data obtained from adults who participated in our standard localizer task that were scanned in BGU. ROIs were defined using the FSL feat package, by employing univariate analysis which is based on the general linear model (GLM) method. ROIs were defined by contrasting BOLD fMRI data as follows: (1) all face conditions (famous faces, unfamiliar faces) vs. places; (2) places vs. all face conditions; and (3) object condition vs. scrambled condition. All contrasts were conducted at a significance level of Z > 2.25 as was previously done in other studies^27^. Finally overlapping voxels between contrasts were excluded. Yet, because the occipital face area (OFA) contained voxels from both face and object selective regions, overlaps between these regions were not excluded^28^. All functional regions were identified with this threshold except for the right inferior temporal sulcus (ITS) that was only evident when applying a lower threshold (Z > 1.5) (see Supplementary Materials—Table S3—Number of voxels within each fROI on the anatomical template space). Next, we projected these ROIs to define ROIs in subjects who only had resting state scans. This was done by the ANTs function “Apply transform” which allows transformation between an individual MRI volume and the average template and vice versa^37^. Then, based on the group contrast, we labeled face, place, and object selective regions manually by ITK-SNAP. Finally, we projected our regions from the common space to the native space of each subject (using the transformation matrices relative to co-registration, and the warping parameters to the template space) (See Fig. 2 for the projection of functional ROIs on the template space).

### Functional connectivity analysis

We examined the functional connectivity between ROIs in different age groups. This was done by conducting pairwise correlation analyses, using the continuous uninterrupted signal as a function of time. We calculated Pearson correlations between all ROIs within and between all regions of the core and extended face selective regions, place selective regions and object selective regions in each subject and run (note that we report only within network connectivity of the core-face selective regions, place selective regions and object selective regions in the main section). We used Fisher transformation so that correlation values could be subjected to statistical parametric testing. In subjects with 2 localizer runs we averaged the data across the two runs. We then calculated the average correlation for each group. While our focus was on the developmental pattern of the face selective regions, to examine the specificity of the findings, we also defined the place and object selective regions as control networks. We calculated the following connectivity measures: connectivity within core face selective regions, connectivity within place selective regions and connectivity within object selective regions separately for each hemisphere. Because our database included participants that were scanned in different sites, we only report within groups effects to avoid any potential confounds that may be related to scanning conditions. Therefore, for each visual category, we compared the functional connectivity within the right hemisphere to the functional connectivity within the left hemisphere. These analyses were carried out based on planned comparisons. In addition, laterality index was calculated separately for each network as follows: (right functional connectivity-left functional connectivity)/(right functional connectivity + left functional connectivity). A positive index indicates right hemispheric preference, while a negative index indicates left hemispheric preference. Note that this index was only used for presentation purposes in this graph and not for statistical analyses.

## Results

### Testing the feasibility of the approach

To validate our method, we first applied it to adults with both localizer and resting state scans. We expected to obtain stronger functional connectivity of the core face selective regions in the right compared to the left hemisphere, as previously reported in the literature^27,35^. Importantly the data from these subjects also provided the ROI definition applied across all age groups for whom only resting scan data were available.

We compared the functional connectivity during the localizer and resting state scans within each visual network, as defined by the localizer scan and compared these patters across hemispheres (see full statistical analyses under Supplementary Materials—Comparing visual and resting state experiments in adults from Rosenthal et al.^30^, Table S4A and B online)*.*

**Figure 3:**
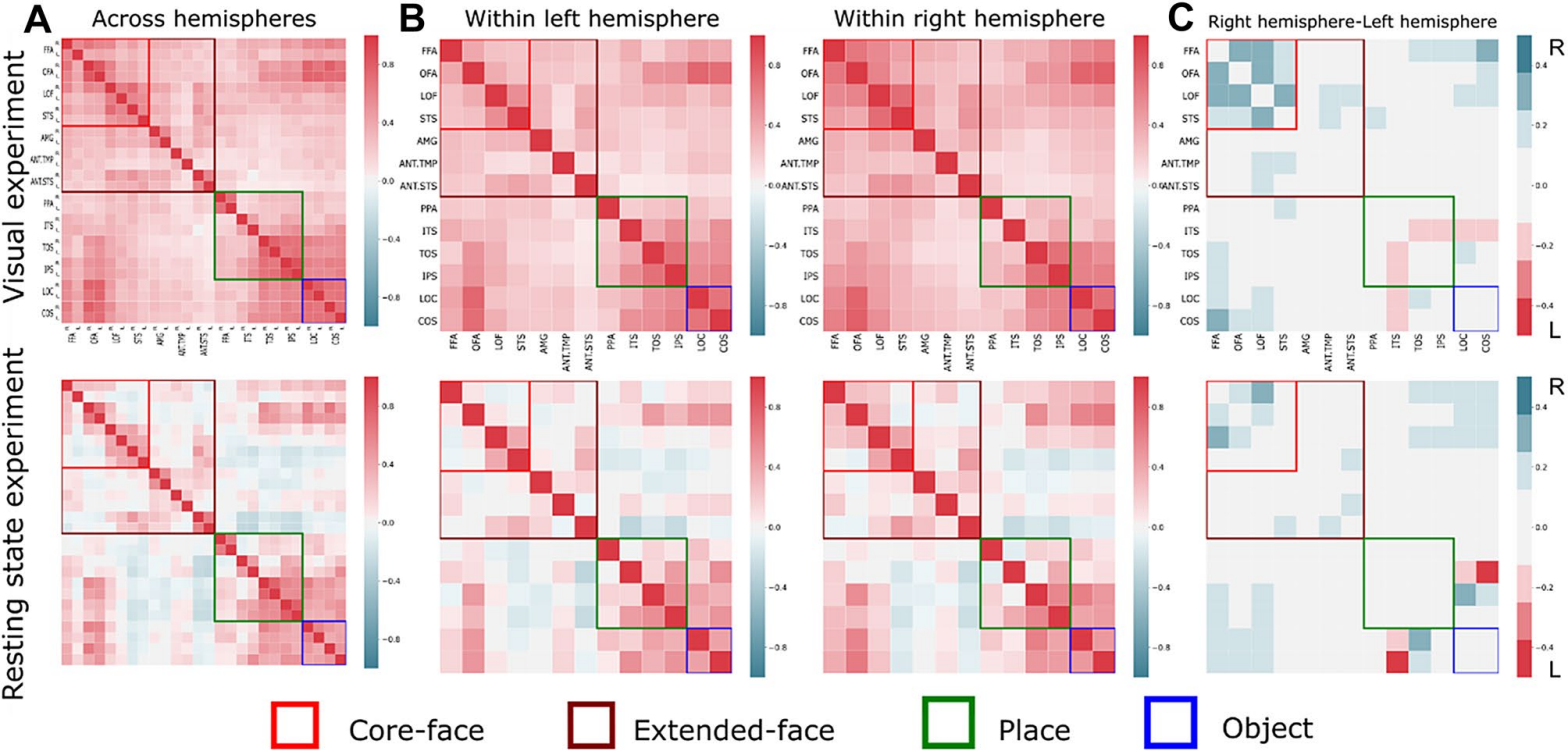
Functional connectivity matrices of adults from Rosenthal et al.^30^. (**A**) Functional connectivity matrices across hemispheres, ROIs names are depicted for the rows and columns and the same convention is used for all panels. (**B**) Functional connectivity matrices within hemispheres. Functional connectivity matrices were obtained from visual (top row) and resting state (bottom row) experiments. Matrices show all pairwise correlations between regions within the core face selective regions (red rectangle), regions in the extended face selective network (brown rectangle), regions that are place selective (green rectangle), and regions that are object selective (blue rectangles). The color code indicates the level of correlation calculated between each pair of regions in each subject and then averaged across groups. (**C**) Matrices depicting the difference in functional connectivity between the right and left hemispheres. Color code ranging from blue, indicating a stronger bias to the right hemisphere to red, indicating a stronger bias to the left hemisphere.

As expected, we observed stronger functional connectivity in the right core-face selective regions compared to the left face selective regions in both the localizer [F (1,19) = 200.100, *p* < 0.000, η^2^_p_ = 0.913] and resting state [F (1,19) = 52.696, *p* < 0.000, η^2^_p_ = 0.735] conditions. Moreover, in the place network, stronger functional connectivity was observed in the left compared to the right hemisphere in the localizer condition [F (1,19) = 5.47, *p* = 0.030, η^2^_p_ = 0.224], while no differences in laterality were observed in the resting state condition [F (1,19) = 1.806, *p* = 0.195, η^2^_p_ = 0.087]. Finally, in the object selective regions, no laterality difference was observed in both localizer and resting state conditions [localizer (F (1,19) = 0.341, *p* = 0.566, η^2^_p_ = 0.018); resting state (F (1,19) = 0.639, *p* = 0.434, η^2^_p_ = 0.032)] (see Fig. 3). Thus, these results attest to the specificity of the right lateralization of the face selective regions.

In the following analysis, we aimed to validate the usage of resting state data in the context of our new approach. Hence, we compared adults’ resting state from the previous analysis and adults from the ABIDE database. These findings would validate that it is possible to use the same approach for other groups for whom obtaining task-related data during scanning is difficult. (see Supplementary Materials—Comparing resting state scans from Rosenthal et al.^30^ and the ABIDE database, Table S5A and B online).

### Cross-sectional comparison of different age groups

**Figure 4:**
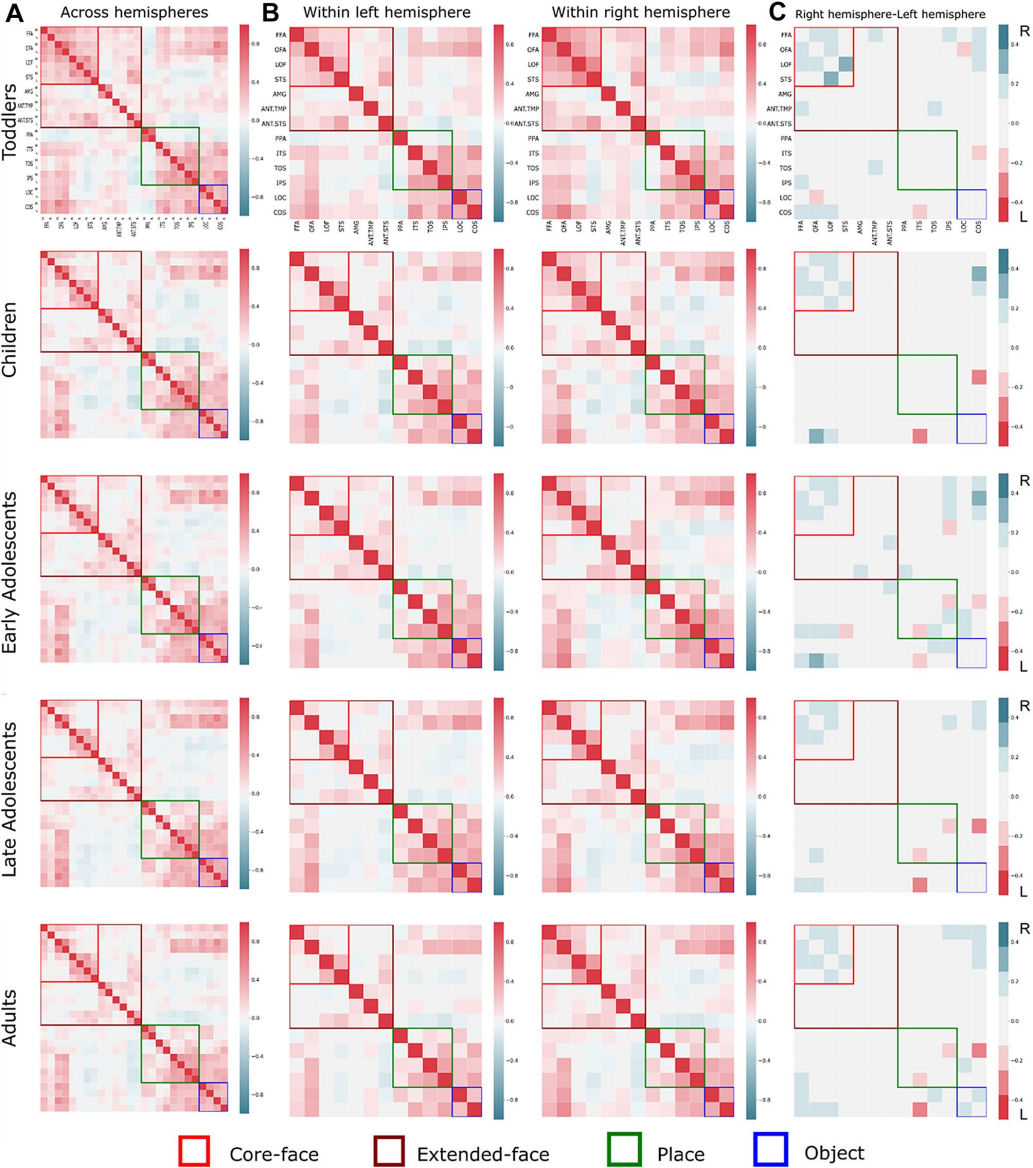
Functional connectivity matrices across development. (**A**) Functional connectivity matrices across hemispheres, ROIs names are depicted for the rows and columns and the same convention is used for all panels. (**B**) Functional connectivity matrices within hemispheres. (**C**) Difference between functional connectivity matrices within hemispheres. All functional connectivity matrices were obtained from resting state scans from toddlers^39^, children, adolescents, and adults^38^. Matrices show all pairwise correlations between regions within the core face selective regions (red rectangle), regions in the extended face selective network (brown rectangle), regions that are place selective (green rectangle), and regions that are object selective (blue rectangles). The color code indicates the level of correlation calculated between each pair of regions in each subject and then averaged across groups. (**C**) Matrices depicting the differences in functional connectivity between the right and left hemispheres.

**Figure 5:**
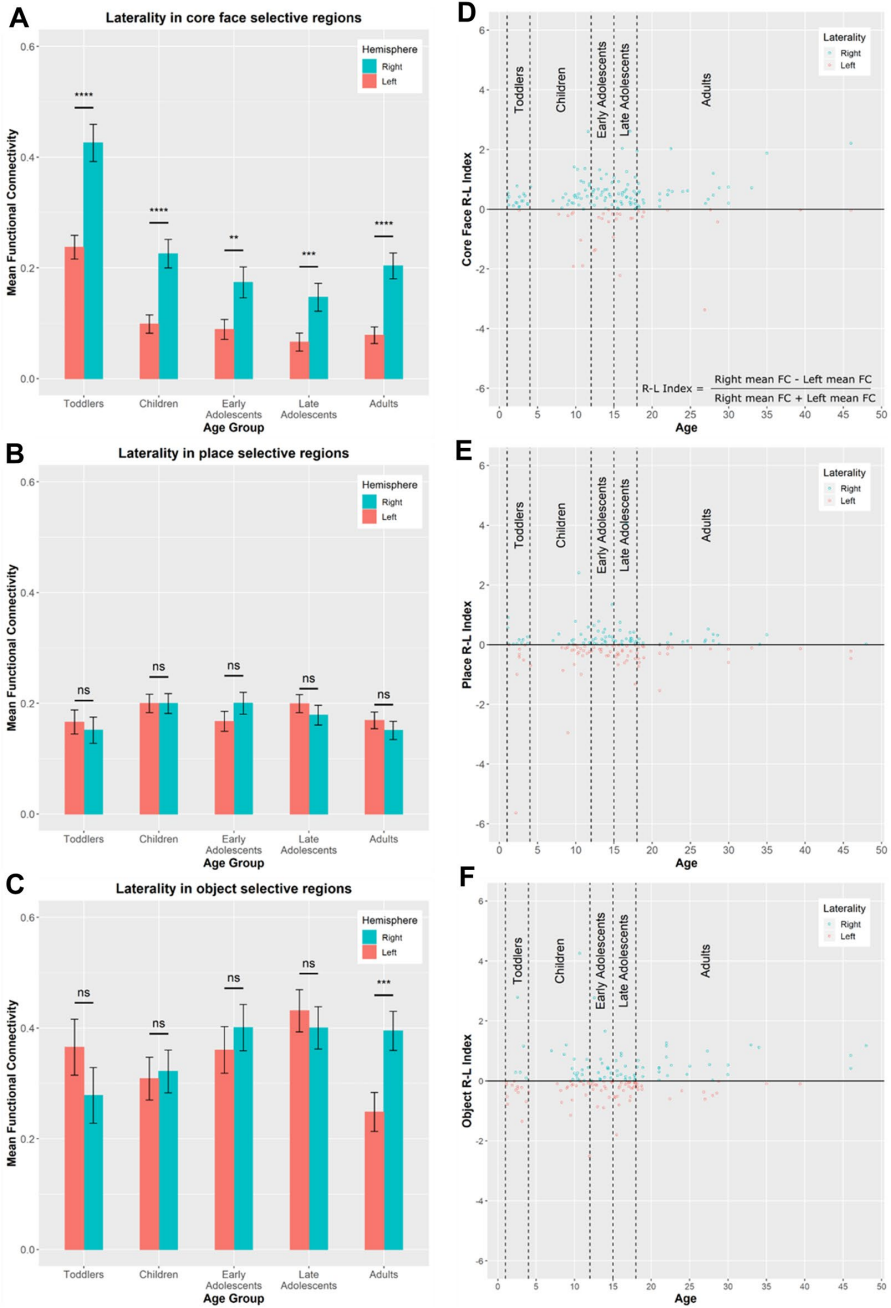
Functional connectivity of the face, place, and object selective regions. Left: mean functional connectivity of the face selective regions in all age groups (**A**) in the place selective regions (**B**) and in the object selective regions (**C**). Right: Graphic depiction of the functional connectivity of all participants within the core face selective regions (**D**), the place selective regions (**E**) and the object selective regions (**F**) using a laterality index, which quantifies the laterality preference in each subject. The more positive the index, the greater the right laterality, and vice versa. Zero indicates no laterality. As is evident, in the mean data (**A**–**C**) and in the individual subjects’ data (**D**–**F**), in the core-face selective regions the functional connectivity was greater in the right than the left hemisphere in all age groups. In the place selective regions, there was no difference between the hemispheres in all age groups. In the object selective regions, in most age groups, there was no difference in laterality between the hemispheres, except for the adult groups that unexpectedly exhibited greater functional connectivity in the right hemisphere.

To validate our method for examining developmental changes, we further tested the specificity of the laterality difference in functional connectivity to the face selective regions by examining the place selective regions and object selective regions using a similar methodology (see more in Figs. 4 and 5; for the rest of the networks see Supplementary Materials—Developmental analysis—hemispheric dominance in the visual networks and Tables S6A–E online). We first conducted a repeated measures ANOVA with the between factors: group (toddlers from Dinstein et al.^39^, children from ABIDE, early adolescents from ABIDE, late adolescents from ABIDE and adults from ABIDE), within factors: network (as described in *Functional connectivity analysis*) and hemisphere. We examined the right hemispheric dominance of the core-face selective regions known from the literature^13^ and that we replicated also in the present study (see Supplementary Materials—Comparing visual and resting state experiments in adults from Rosenthal et al.^30^, Fig. 3 and Supplementary Materials—Comparing resting state scans from Rosenthal et al.^30^ and the ABIDE database). The matrices showing these correlations are presented in Fig. 4. We did not find a main effect of group (F (4,154) = 2.105, *p* = 0.083, η^2^_p_ = 0.052), but did find a main effect of network (F (7,1078) = 160.920, *p* < 0.000, η^2^_p_ = 0.511) and an interaction between these factors (F (28,1078) = 4.832, *p* < 0.000, η^2^_p_ = 0.222). We also found a main effect of hemisphere (F (1,154) = 44.005, *p* < 0.000, η^2^_p_ = 0.222), and no interaction between hemisphere and group (F (4,154) = 1.668, *p* = 0.160, η^2^_p_ = 0.041). We found an interaction between network and hemisphere (F (7,1078) = 16.661, *p* < 0.000, η^2^_p_ = 0.098). Finally, we found an interaction between network, hemisphere, and group (F (28,1078) = 2.342, *p* < 0.000, η^2^_p_ = 0.057).

To examine the hemispheric dominance of each network in each age group, we compared the right and the left functional connectivity (see more in Tables S4A–E). The connectivity of the core-face selective regions was larger in the right compared to the left hemisphere in all age groups. Within core-face connectivity, as expected, we found greater connectivity in the right hemisphere than the left in adults (F (1,154) = 33.277, *p* < 0.000, η^2^_p_ = 0.178). Importantly, a similar right dominance was also evident in toddlers (F (1,154) = 36.834, *p* < 0.000, η^2^_p_ = 0.193), children (F (1,154) = 28.461, *p* < 0.000, η^2^_p_ = 0.156), early adolescents (F (1,154) = 10.901, *p* = 0.001, η^2^_p_ = 0.067) and late adolescents (F (1,154) = 11.876, *p* = 0.0007, η^2^_p_ = 0.072).

In contrast, within the place selective regions, the functional connectivity did not differ significantly across hemispheres in toddlers (F (1,154) = 0.385, *p* = 0.536, η^2^_p_ = 0.002), children (F (1,154) = 0.000, *p* = 0.999, η^2^_p_ = 0.000), early adolescents (F (1,154) = 2.826, *p* = 0.095, η^2^_p_ = 0.018), late adolescents (F (1,154) = 1.338, *p* = 0.249, η^2^_p_ = 0.009) and adults (F (1,154) = 1.207, *p* = 0.274, η^2^_p_ = 0.008). Within the object selective regions, no hemispheric differences were found in toddlers (F (1,154) = 2.069, *p* = 0.152, η^2^_p_ = 0.013), children (F (1,154) = 0.080, *p* = 0.778, η^2^_p_ = 0.0005), early adolescents (F (1,154) = 0.644, *p* = 0.423, η^2^_p_ = 0.004) and late adolescents (F (1,154) = 0.450, *p* = 0.503, η^2^_p_ = 0.003). Unexpectedly, in the adult group from the ABIDE dataset, there was greater connectivity in the right compared to the left hemisphere (F (1,154) = 12.137, *p* = 0.0006, η^2^_p_ = 0.07). We note that the functional connectivity obtained for objects is based on connectivity between only two regions (LOC, COS) in each hemisphere, therefore this measure is less reliable. In addition, this group exhibited a significantly larger effect size in the core-face selective regions compared to the laterality of the object selective regions, which suggests that in adults, the right laterality of the core-face selective regions is a more pronounced effect (see Fig. 5 and Supplementary Materials Tables S6A–E online). In an analysis of an independent dataset that included groups of children, adolescents and adults from ABIDE2^45^**,** we replicated the main findings of the right laterality of the functional connectivity in the core-face selective regions in all age groups. In contrast, no right dominance of the functional connectivity was found within the place and the object selective regions in all age groups (children, adolescents, and adults). This analysis supports the main findings reported above. (For more details of this analyses see Supplementary Materials—Developmental analysis—hemispheric dominance in the visual networks, Control analyses—1. Analysis of an independent dataset based on ABIDE2 and Table S8A–C).

To further validate the results showing the right laterality of the functional connectivity in core face regions, we compared the quality of the BOLD signal across the two hemispheres using time series signal-to-noise ratio (tSNR). This measurement was shown to provide an indication of the quality of fMRI data that might in turn affect functional connectivity^46,47^. We compared the tSNR between the right and the left core-face selective regions and found no significant differences in all age groups. This result demonstrates that the tSNR cannot account for the right laterality of the functional connectivity of the core-face selective regions (see details of the analysis in Supplementary Materials—Control analyses—Time series signal-to-noise ratio (tSNR) analysis and Table S9A,B online).

Finally, to further probe into the results of the right laterality, we wanted to examine which specific edges connecting fROIs within the main visual categories (core-faces, places, and objects) contributed the most to this finding. To do so, we used a linear discrimination analysis (LDA)^48^ and participants’ age and the functional connectivity between the fROIS of the visual selective regions (core-face, places and objects) were defined as classifiers of the right and left hemisphere.

Importantly, age had the lowest absolute value, suggesting that this feature shows the least contribution in predicting the hemispheric laterality. This further confirms our main result of right lateralization in all age groups. When examining the contribution of specific edges, we found that, within the core-face selective regions, the functional connectivity between the FFA and LOF, FFA and OFA, OFA and LOF, and LOF and pSTS had a positive and the highest absolute values. This suggests that these functional connections contributed the most to predicting the right hemispheric laterality. This result is consistent with the known role of these regions in face processing^27^. (See more details in Supplementary Materials—Assessing the contribution of different fROIs to the right lateralization of face functional connectivity and Table S10 online).

## Discussion

In this study we investigated the typical developmental trajectory of the functional connectivity of the face selective regions from infancy to adulthood. Our results demonstrate that core face processing brain areas exhibit right hemisphere dominance from the first years of life^2,15,21^. This dominance was not apparent in object or place processing regions of infants or toddlers, suggesting early rightward lateralization as previously reported for adults^11–13^.

One of the challenges in investigating such developmental changes is the difficulty to acquire task-related scans from toddlers or young children which would allow delineating regions of interests (but see^2,15^ for task-fMRI in young babies). Additionally, analyzing data from different age groups requires standardization of the brain anatomy, a procedure which usually involves normalization relative to an adult brain and might introduce deformations. To address these challenges, we implemented an alternative approach that is based on mathematically merging the brains of all subjects in the study to a single template brain and registering every subject to that common space. Moreover, functional ROIs of the face, place and object selective regions were based on data obtained from adults that were scanned with a localizer experiment and were then projected to all other participants using the template approach we applied in this study.

Right laterality of face selective regions was previously documented already in newborns in ERP studies^22^. fMRI studies found that children age 5–8 years old showed more bilateral responses to faces, while adolescents exhibited patterns that were more adult-like, suggesting a graded trajectory of this organization, this pattern was evident in both face selectivity and volumes of the face-selective regions^23,25^. Yet, functional connectivity provides a different, important facet of the development of the face system that has been less studied so far.

Based on the Interactive Specialization (IS) model^32^, cortical specialization develops from the interaction between activity in different regions. This assertion was supported by findings showing that anatomical connectivity can predict functional selectivity^49^. Moreover, it has been reported that early anatomical connectivity (at age 5 years old) can predict later function in the visual word form area^50^. Therefore, early functional connectivity might be an important property of the development of face processing specialization. Yet, this aspect was mainly investigated in adults^27,30^, adolescents and children^33–35^ and only lately in toddlers^21^. This latter study reported an early specific property of the development of the face network such that it exhibited connectivity to foveal V1, while the place network exhibited connectivity with peripheral V1. Our findings reveal that the known right hemispheric dominance of the face network which exists in adults’ functional connectivity^13^, is already evident from infancy. Moreover, this lateralization is specific to faces and not to other categories such as objects or places. Importantly, the edges that contributed the most to this result were the edges between the right core-face selective regions. In addition, this pattern was stable and replicable in another database and could not be attributed to differences in signal quality across hemispheres as quantified by tSNR in the core-faces selective regions.

Previous studies focused on the development of the right lateralization of face selectivity imply that such lateralization develops in tandem with literacy acquisition. Specifically, initially bilateral language regions in ventral occipital temporal cortex are involved in literacy acquisition but the language system becomes more left lateralized as these visual regions are gradually associated with other language regions. Hence, as a result of this left lateralization, face representations become more right lateralized^11,23^. Additional studies also show that the increase in face and word selectivity were associated with a decrease in body-part selectivity, thus showing that selectivity in ventral temporal cortex is repurposed into word and face selectivity during child development^18^.

These patterns suggest some cortical recycling that involves face, language and body selectivity during development^18^. Together these findings imply that the right laterality of functional connectivity in the core-face selective regions that was found in our study may be an important feature that would later enable the cortical specialization of face processing that continues to change during development^11^. Future studies should investigate and compare the trajectory of the laterality of the functional connectivity of face selective regions to these of the language and body selective regions and relate such findings to the behavioral measures of their processing to further investigate the specificity of these mechanisms.

## Limitations

This study was based on a cross-sectional, rather than a longitudinal design and hence could not follow the development of visual system organization in the same individuals over time. Moreover, we employed data from different scanning sites and included resting state scans with different scanning settings and parameters that can potentially impact functional connectivity magnitudes. To overcome this source of variability, we performed all comparisons of functional connectivity within group (Figs. 4, 5). This demonstrates the reliability of findings across sites. Another limitation of the current study was the definition of functional ROIs based on a sub-group of adults rather than individually in each of the subjects participating in the study. Given the known variability in the location of functional face related ROI^51^, this procedure might be less precise compared to individually defined ROIs.

Given these limitations, in future studies, it would be important to replicate and extend our findings by investigating and comparing changes in the functional connectivity of the face selective regions within and between hemispheres between different age groups. In addition, previous studies revealed associations between hemispheric laterality and different aspects of face-processing, such as holistic processing^52^. Therefore, future studies should also investigate the relation between such behavioral characteristics and the changes occurring in that system at the neural level. Such behavioral measures, however, would only be possible to obtain in participants who are old enough to perform such tasks. In addition, this should also be carried out by including participants that were scanned under the same conditions with similar group size across all age groups in cross-sectional and ideally also longitudinal designs.

## Conclusions

The current study revealed that core face selective regions are lateralized from early stages of development as evident in stronger functional connectivity in the right hemisphere. This highlights the uniqueness of the face processing network and suggests that early defined anatomical and functional structures induce a lateralized organization that is evident in early functional activity even during resting state conditions.

### Supplementary Information

Supplementary Information.

## Data Availability

Data will be made available upon request to the authors.
