# Supplementary material for: Functional connectivity of the human face network exhibits right hemispheric lateralization from infancy to adulthood
Source: Sci Rep. 2023 Nov 27;13:20831. doi: 10.1038/s41598-023-47581-z (PMC10682179; doi:10.1038/s41598-023-47581-z)
Supplement: Supplementary file 1 — Supplementary Information. [file 41598_2023_47581_MOESM1_ESM.pdf]

# Supplementary Materials

## Methods

### *Table S1- Site parameters and subject inclusion*

| Database | Site | Source | Tesla | TR | Volumes before scrubbing | Total subjects before scrubbing | Subjects remaining after scrubbing (>5.5 minutes) | Subjects filtered for SNR | Subjects included for resting state analysis | Subjects included in the template |
|---|---|---|---|---|---|---|---|---|---|---|
| ABIDE1 | NYU | Di Martino et al. [37] | 3 | 2 | 180 | 105 | 38 | 38 | 0 | 0 |
| ABIDE1 | UM | Di Martino et al. . [37] | 3 | 2 | 300 | 77 | 55 | 0 | 55 | 55 |
| ABIDE1 | Yale | Di Martino et al. . [37] | 3 | 2 | 200 | 28 | 8 | 0 | 8 | 8 |
| ABIDE1 | CMU | Di Martino et al. . [37] | 3 | 2 | 240 | 13 | 3 | 0 | 3 | 3 |
| ABIDE1 | KKI | Di Martino et al. . [37] | 3 | 2.5 | 156 | 33 | 3 | 0 | 3 | 3 |
| ABIDE1 | MaxMun | Di Martino et al. . [37] | 3 | 3 | 120 | 33 | 11 | 0 | 11 | 11 |
| ABIDE1 | SDSU | Di Martino et al. . [37] | 3 | 2 | 180 | 22 | 7 | 0 | 7 | 7 |
| ABIDE1 | Stanford | Di Martino et al. . [37] | 3 | 2 | 240 | 20 | 10 | 0 | 10 | 10 |
| ABIDE1 | OHSU | Di Martino et al. . [37] | 3 | 2.5 | 82 | 15 | 0 | 0 | 0 | 0 |
| ABIDE1 | SBL | Di Martino et al. . [37] | 3 | 2.2 | 200 | 15 | 0 | 0 | 0 | 0 |
| ABIDE1 | Trinity | Di Martino et al. . [37] | 3 | 2 | 150 | 25 | 0 | 0 | 0 | 0 |
| ABIDE1 | OLIN | Di Martino et al. . [37] | 3 | 1.5 | 210 | 16 | 0 | 0 | 0 | 0 |
| ABIDE1 | UCLA | Di Martino et al. . [37] | 3 | 3 | 120 | 47 | 11 | 0 | 11 | 11 |
| ABIDE1 | PITT | Di Martino et al. . [37] | 3 | 1.5 | 200 | 27 | 0 | 0 | 0 | 0 |
| ABIDE1 | Leuven | Di Martino et al. . [37] | 3 | 1.67872 | 250 | 35 | 8 | 0 | 8 | 8 |
| ABIDE1 | USM | Di Martino et al. . [37] | 3 | 2 | 240 | 43 | 23 | 0 | 23 | 23 |
| ABIDE1 | Caltech | Di Martino et al. . [37] | 3 | 2 | 150 | 19 | 0 | 0 | 0 | 0 |
| SD | SD | Dinstein et al. [38] | 1.5 | 2.5 | 154 | 29 | 20 | 0 | 20 | 20 |
| BGU | BGU | Rosenthal et al. [29] | 3 | 2 | 292/225 | 35 | 20 | 0 | 20 | 35 |
| **Total** | | | | | | **637** | **217** | **38** | **179** | **194** |

### *Table S2 – Instructions given to participants in each resting state experiment in each site:*

| Site | Source | Resting state procedure |
|---|---|---|
| NYU | Di Martino et al. [37] | Eyes open, while a white cross-hair against a black background was projected on a screen. |
| UM | Di Martino et al. [37] | Eyes-open, while fixating a cross in the middle of the screen. |
| Yale | Di Martino et al. [37] | Eyes open, while black background with a gray central fixation cross was presented. |
| CMU | Di Martino et al. [37] | Eyes closed, while the room lights were shut off. |
| KKI | Di Martino et al. [37] | Fixating a crosshair on a black computer screen. |
| MaxMun | Di Martino et al. [37] | Eyes closed while no visual stimulus was presented or eyes open and looking at a picture of a night sky with stars. |
| SDSU | Di Martino et al. [37] | Eyes open, fixating a cross, trying not to fall asleep. |
| Stanford | Di Martino et al. [37] | Eyes closed. |
| OHSU | Di Martino et al. [37] | Participants were scanned with eyes open in three sequential scans. |
| SBL | Di Martino et al. [37] | Eyes closed. |
| Trinity | Di Martino et al. [37] | Eyes closed. |
| OLIN | Di Martino et al. [37] | Eyes open, fixating a cross |

| | | |
|---|---|---|
| UCLA | Di Martino et al. [37] | Eyes open, while a white screen with a black fixation cross in the middle of the screen was presented |
| PITT | Di Martino et al. [37] | Closed eyes, while not falling asleep. |
| Leuven | Di Martino et al. [37] | Fixating on a white cross against a black background. |
| USM | Di Martino et al. [37] | Eyes open without focusing on any particular mental activity. |
| Caltech | Di Martino et al. [37] | Eyes closed, while staying awake. |
| SD | Dinstein et al. [38] | Natural sleep |
| BGU | Rosenthal et al. [29] | Viewing a plain black screen |

*Table S3 – Number of voxels within each fROI on the anatomical template space:*

| fROI | Number of voxels |
|---|---|
| FFA_R | 2418 |
| FFA_L | 799 |
| OFA_R | 4578 |
| OFA_L | 2094 |
| LOF_R | 3253 |
| LOF_L | 1129 |
| pSTS_R | 3663 |
| pSTS_L | 1230 |
| AMG_R | 1539 |
| AMG_L | 1395 |
| ANT.TMP_R | 452 |
| ANT.TMP_L | 334 |
| ANT.STS_R | 1232 |
| ANT.STS_L | 1231 |
| PPA_R | 2014 |
| PPA_L | 1247 |
| ITS_R | 332 |
| ITS_L | 711 |
| TOS_R | 1236 |
| TOS_L | 1125 |
| IPS_R | 2840 |
| IPS_L | 2727 |
| LOC_R | 2109 |
| LOC_L | 1943 |
| COS_R | 1527 |
| COS_L | 941 |

**Results**

**Comparing visual and resting state experiments in adults from Rosenthal et al. [29]**

We performed a repeated measures ANOVA– within-subject factors – scan, network (as described in *Functional connectivity analysis*), and hemisphere. Face selective regions exhibited main effects for scan ($F(1,19) = 386.353$, $p < 0.0000$, $\eta^2_p = 0.953$), network ($F(1,19) = 138.189$, $p < 0.0000$, $\eta^2_p = 0.879$) and hemisphere ($F(1,19) = 31.656$, $p < 0.0001$, $\eta^2_p = 0.625$). The ANOVA also revealed an interaction between scan and network ($F(7,133) = 15.518$, $p < 0.0000$, $\eta^2_p = 0.449$), but no interaction was found between scan and hemisphere ($F(1,19) = 2.491$, $p=0.131$, $\eta^2_p = 0.116$). As expected, we found an interaction between hemisphere and network ($F(7,133) = 11.017$, $p < 0.0000$, $\eta^2_p = 0.367$). In addition, there was a 3-way interaction between scan, hemisphere, and network ($F(7,133) = 2.976$, $p=0.006$, $\eta^2_p = 0.135$). Therefore, we compared the connectivity of the right and left hemispheres in each network (see more *Tables S4A* and *S4B*). The main networks we focused on, the core face regions, the place selective regions and the object selective regions, replicated the expected pattern during both the visual and the resting state experiment such that the within face connectivity in the core face ROIs exhibited right dominance. The connectivity within the place selective regions was greater in the left hemisphere in the visual experiment condition, while no hemispheric difference was found in the resting state condition. In the object selective regions, no difference was found between the hemispheres in both scanning conditions. Our focus was the hemispheric differences in the functional connectivity within the main visual networks (core face, place and object) and not in the extended face network and between the visual network, yet we included these data in our analysis (see *Tables S4A* and *S4B*).

Importantly, this pattern of results replicates the organization of the face selective regions expected from previous studies [13, 27] using standard analysis approaches and hence validates the new methodology developed in the present study.

Table S4A: Visual experiment condition - Comparison of functional connectivity across hemispheres in each network.

Significant results are indicated in bold.

| Network connectivity | | Mean Right | Mean Left | Right-Left contrast | | | |
|---|---|---|---|---|---|---|---|
| | | | | F | DF | p | $\eta^2_p$ |
| Within face connectivity | **1. Core** | **0.656** | 0.370 | **200.100** | **1,19** | **0.000** | **0.913** |
| | **2. extended** | **0.288** | 0.209 | **5.170** | **1,19** | **0.035** | **0.214** |
| | **3. Between core and extended** | **0.350** | 0.265 | **17.882** | **1,19** | **0.0004** | **0.485** |
| **Within place connectivity** | | 0.378 | **0.440** | **5.473** | **1,19** | **0.030** | **0.224** |
| **Between core and place connectivity** | | **0.362** | 0.304 | **16.133** | **1,19** | **0.0007** | **0.459** |
| Within object connectivity | | 0.865 | 0.833 | 0.341 | 1,19 | 0.566 | 0.018 |
| **Between core and object connectivity** | | **0.648** | 0.476 | **45.851** | **1,19** | **0.000** | **0.707** |
| Between place and object connectivity | | 0.467 | 0.485 | 0.676 | 1,19 | 0.421 | 0.034 |

Table S4B: Resting state condition - Comparison of functional connectivity across hemispheres in each network.

Significant results are indicated in bold.

| Network connectivity | | Mean Right | Mean Left | Right-Left contrast | | | |
|---|---|---|---|---|---|---|---|
| | | | | F | DF | p | $\eta^2_p$ |
| Within face connectivity | **1. Core** | **0.192** | 0.067 | **52.696** | **1,19** | **0.000** | **0.735** |
| | **2. Extended** | **0.170** | 0.076 | **9.563** | **1,19** | **0.006** | **0.335** |
| | 3. Between core and extended | 0.088 | 0.059 | 1.719 | 1,19 | 0.205 | 0.083 |
| Within place connectivity | | 0.218 | 0.245 | 1.806 | 1,19 | 0.195 | 0.087 |
| **Between core and place connectivity** | | **0.096** | 0.041 | **10.043** | **1,19** | **0.005** | **0.346** |
| Within object connectivity | | 0.525 | 0.463 | 0.639 | 1,19 | 0.434 | 0.032 |
| **Between core and object connectivity** | | **0.291** | 0.168 | **24.976** | **1,19** | **0.0001** | **0.568** |
| Between place and object connectivity | | 0.331 | 0.352 | 1.230 | 1,19 | 0.281 | 0.061 |

**Comparing resting state scans from Rosenthal et al. [29] and the ABIDE database**

Our next goal was to use our newly developed methodology on subjects that could not perform a task during scanning and were only scanned during resting state and hence did not have their own ROI definition. Specifically, in the following analysis, we aimed to validate the usage of resting state data and we therefore compared between adults' resting state from the previous stage (see *Supplementary Information - Comparing visual and resting state experiments in adults from Rosenthal et al.* [29] and adults from the ABIDE database.

We performed a repeated measures ANOVA between factors: group (Rosenthal et al. [29]/ ABIDE), within factors: network (as described in *Functional connectivity analysis*) and hemisphere. Similarly, to the analysis performed in the adults from Rosenthal et al. [29] that included a visual experiment, we wanted to identify the organization of the face selective regions in the ABIDE group and hence, we conducted a second analysis with hemisphere as another within subject factor. We found a main effect of group (F (1,59) = 19.308, $p<0.0001$, $\eta^2_p = 0.246$) and network (F (7,413) = 84.911, $p<0.000001$, $\eta^2_p = 0.590$) and an interaction between these factors (F (7, 413) = 5.842, $p<0.00001$, $\eta^2_p = 0.090$). A main effect was found for hemisphere (F (1,59) = 45.744, $p<0.000001$, $\eta^2_p = 0.437$) and no interaction between hemisphere and group (F (1,59) =0.005, $p=0.944$, $\eta^2_p = 0$). As expected, we found an interaction between network and hemisphere (F (7,413) =9.019, $p<0.000001$, $\eta^2_p = 0.133$). There was no interaction between network, hemisphere, and group (F (7, 413) =1.246, $p=0.276$, $\eta^2_p = 0.021$). Hence, we compared the between connectivity in each hemisphere and network (*Tables S5* A & B). As expected, and described above, in the adult group that participated in both the visual and resting state scans, the core face selective regions exhibited right dominance in both groups of adults and no difference in hemispheric connectivity within the connectivity of the place network in both groups. In the object selective regions, no hemispheric difference was found in the adult group from Rosenthal et al. [29], while the adults from the ABIDE dataset exhibited right dominance. Yet, the effect sizes of the functional connectivity in core face selective regions were larger compared to the object selective regions in the group of ABIDE adults. In addition, object selective regions within each hemisphere were based only on one edge and hence might be unstable. These findings validate

that it is possible to use the same approach for other groups for whom obtaining task-related data during scanning is difficult or even impossible such as toddlers.

The tables below also include analyses of the hemispheric differences in the functional connectivity within the visual networks and between the visual network and within the extended face network (see *Tables S5A* and *S5B*).

Table S5A: Rosenthal et al. [29] – Comparison of the connectivity in each hemisphere in each network

Significant results are indicated in bold.

| Network connectivity | | Mean Right | Mean Left | Right-Left contrast | | | |
|---|---|---|---|---|---|---|---|
| | | | | F | DF | p | $\eta^2_p$ |
| Within face connectivity | **1. Core** | **0.192** | 0.067 | **18.946** | **1,59** | **0.000** | **0.243** |
| | **2. Extended** | **0.170** | 0.076 | **11.070** | **1,59** | **0.001** | **0.158** |
| | 3. Between core and extended | 0.088 | 0.059 | 2.565 | 1,59 | 0.115 | 0.0417 |
| Within place connectivity | | 0.218 | 0.245 | 2.151 | 1,59 | 0.148 | 0.035 |
| **Between core and place connectivity** | | **0.096** | 0.041 | **10.306** | **1,59** | **0.002** | **0.149** |
| Within object connectivity | | 0.525 | 0.463 | 0.882 | 1,59 | 0.351 | 0.015 |
| **Between core and object connectivity** | | **0.291** | 0.168 | **27.162** | **1,59** | **0.000** | **0.315** |
| Between place and object connectivity | | 0.331 | 0.352 | 0.819 | 1,59 | 0.369 | 0.014 |

Table S5B: ABIDE – Comparison of the connectivity in each hemisphere in each network

Significant results are indicated in bold.

| Network connectivity | | Mean Right | Mean Left | Right-Left contrast | | | |
|---|---|---|---|---|---|---|---|
| | | | | F | DF | p | $\eta^2_p$ |
| Within face connectivity | **1. Core** | **0.203** | 0.078 | **38.742** | **1,59** | **0.000** | **0.396** |
| | 2. Extended | 0.121 | 0.083 | 3.605 | 1,59 | 0.062 | 0.058 |
| | 3. Between core and extended | 0.047 | 0.028 | 2.239 | 1,59 | 0.140 | 0.037 |
| Within place connectivity | | 0.151 | 0.169 | 1.920 | 1,59 | 0.171 | 0.031 |
| **Between core and place connectivity** | | **0.075** | 0.051 | **4.101** | **1,59** | **0.047** | **0.065** |
| **Within object connectivity** | | **0.395** | 0.248 | **10.026** | **1,59** | **0.002** | **0.145** |
| **Between core and object connectivity** | | **0.212** | 0.112 | **36.201** | **1,59** | **0.000** | **0.380** |
| Between place and object connectivity | | 0.219 | 0.221 | 0.017 | 1,59 | 0.898 | 0.0003 |

**Developmental analysis - hemispheric dominance in the visual networks**

We examined the connectivity in an infant group that was tested in Dinstein et al. [38], as well as children, early adolescents, late adolescents, and adult groups from the ABIDE database and cross-sectionally examined the changes in the connectivity patterns across age. We first conducted a repeated measures ANOVA with the between factors: group (toddlers from Dinstein et al. [38], children from ABIDE, early adolescents from ABIDE, late adolescents from ABIDE and adults from ABIDE), within factors: network (as described in *Functional connectivity analysis*) and hemisphere. We examined the right hemispheric dominance of the core-face selective regions known from the literature [13] and that we replicated also in the present study (see *Supplementary- Comparing visual and resting state experiments in adults from Rosenthal et al.* [29]*, Figure 3* & *Supplementary – comparing resting state scans from* Rosenthal et al.[29] *and the ABIDE database*). The matrices showing these correlations are presented in *Figure 4*. We did not find a main effect of group ($F(4,154) = 2.105$, $p=0.083$, $\eta^2_p=0.052$), but did find a main effect of network ($F(7,1078) = 160.920$, $p<0.000$, $\eta^2_p=0.511$) and an interaction between these factors ($F(28,1078) = 4.832$, $p<0.000$, $\eta^2_p=0.222$). We also found a main effect of hemisphere ($F(1,154) = 44.005$, $p<0.000$, $\eta^2_p=0.222$), and no interaction between hemisphere and group ($F(4,154) = 1.668$, $p=0.160$, $\eta^2_p=0.041$). We found an interaction between network and hemisphere ($F(7,1078) = 16.661$, $p<0.000$, $\eta^2_p=0.098$). Finally, we found an interaction between network, hemisphere, and group ($F(28,1078) = 2.342$, $p<0.000$, $\eta^2_p=0.057$).

To examine the hemispheric dominance of each network in each age group, for each network in each age group we compared the right and the left functional connectivity (see more in *Tables* S6A-E). The connectivity of the core-face selective regions was larger in the right compared to the left hemisphere in all age groups. While the within place connectivity exhibited no hemispheric differences in the other age groups. Within the object selective regions, all age groups exhibited no hemispheric difference except for the adults' group that showed larger connectivity in the right hemisphere. The tables below also include analysis of hemispheric differences in the functional connectivity within the visual networks and between the visual network and within the extended face network (see *Tables S6A - E*).

This pattern of results suggests that the core-face selective regions have an early right biased organization which is specific to faces as the organization differs from the organization of the place and object selective regions.

Table S6A: Toddlers – Comparison of the connectivity in each hemisphere in each network

Significant results are indicated in bold.

| Network connectivity | | Mean Right | Mean Left | Right-Left contrast | | | |
|---|---|---|---|---|---|---|---|
| | | | | F | DF | p | $\eta^2_p$ |
| Within face connectivity | **1. Core** | **0.426** | 0.237 | **36.834** | **1,154** | **0.0000** | **0.193** |
| | 2. Extended | 0.058 | 0.044 | 0.186 | 1,154 | 0.666 | 0.001 |
| | **3. Between core and extended** | **0.129** | 0.092 | **4.509** | **1,154** | **0.035** | **0.028** |
| Within place connectivity | | 0.151 | 0.166 | 0.385 | 1,154 | 0.536 | 0.002 |
| Between core and place connectivity | | 0.072 | 0.072 | 0.001 | 1,154 | 0.980 | 0.000 |
| Within object connectivity | | 0.278 | 0.365 | 2.069 | 1,154 | 0.152 | 0.013 |
| **Between core and object connectivity** | | **0.248** | 0.194 | **4.572** | **1,154** | **0.034** | **0.029** |
| Between place and object connectivity | | 0.241 | 0.241 | 0.0001 | 1,154 | 0.991 | 0.000 |

Table S6B: Children – Comparison of the connectivity in each hemisphere in each network

Significant results are indicated in bold.

| Network connectivity | | Mean Right | Mean Left | Right-Left contrast | | | |
|---|---|---|---|---|---|---|---|
| | | | | F | DF | p | $\eta^2_p$ |
| Within face connectivity | **1. Core** | **0.225** | 0.098 | **28.461** | **1,154** | **0.0000** | **0.156** |
| | 2. extended | 0.113 | 0.095 | 0.540 | 1,154 | 0.463 | 0.003 |
| | 3. Between core and extended | 0.056 | 0.042 | 1.070 | 1,154 | 0.303 | 0.007 |
| Within place connectivity | | 0.200 | 0.200 | 0.000 | 1,154 | 0.999 | 0.000 |
| Between core and place connectivity | | 0.059 | 0.040 | 1.801 | 1,154 | 0.182 | 0.011 |
| Within object connectivity | | 0.321 | 0.308 | 0.080 | 1,154 | 0.778 | 0.0005 |
| **Between core and object connectivity** | | **0.240** | 0.152 | **21.083** | **1,154** | **0.0000** | **0.120** |
| Between place and object connectivity | | 0.226 | 0.240 | 0.515 | 1,154 | 0.474 | 0.003 |

Table S6C: Early adolescents – Comparison of the connectivity in each hemisphere in each network

Significant results are indicated in bold.

| Network connectivity | | Mean Right | Mean Left | Right-Left contrast | | | |
|---|---|---|---|---|---|---|---|
| | | | | F | DF | p | $\eta^2_p$ |
| | **1. core** | **0.174** | 0.089 | **10.901** | **1,154** | **0.001** | **0.067** |

| | | | | | | | |
|---|---|---|---|---|---|---|---|
| Within face connectivity | 2. extended | 0.180 | 0.130 | 3.514 | 1,154 | 0.063 | 0.022 |
| | 3. Between core and extended | 0.029 | 0.042 | 0.705 | 1,154 | 0.402 | 0.004 |
| Within place connectivity | | 0.200 | 0.167 | 2.826 | 1,154 | 0.095 | 0.018 |
| **Between core and place connectivity** | | **0.094** | 0.047 | **9.575** | **1,154** | **0.002** | **0.058** |
| Within object connectivity | | 0.400 | 0.360 | 0.644 | 1,154 | 0.423 | 0.004 |
| **Between core and object connectivity** | | **0.230** | 0.138 | **19.332** | **1,154** | **0.000** | **0.111** |
| Between place and object connectivity | | 0.248 | 0.269 | 0.909 | 1,154 | 0.342 | 0.006 |

Table S6D: Late adolescents – Comparison of the connectivity in each hemisphere in each network

Significant results are indicated in bold.

| Network connectivity | | Mean Right | Mean Left | Right-Left contrast | | | |
|---|---|---|---|---|---|---|---|
| | | | | F | DF | p | $\eta^2_p$ |
| Within face connectivity | **1. Core** | **0.147** | 0.066 | **11.876** | **1,154** | **0.0007** | **0.072** |
| | **2. Extended** | **0.127** | 0.054 | **9.174** | **1,154** | **0.003** | **0.056** |
| | 3. Between core and extended | 0.050 | 0.027 | 2.775 | 1,154 | 0.098 | 0.018 |
| Within place connectivity | | 0.179 | 0.199 | 1.338 | 1,154 | 0.249 | 0.009 |
| Between core and place connectivity | | 0.081 | 0.070 | 0.715 | 1,154 | 0.399 | 0.005 |
| Within object connectivity | | 0.400 | 0.431 | 0.450 | 1,154 | 0.503 | 0.003 |
| **Between core and object connectivity** | | **0.206** | 0.141 | **11.436** | **1,154** | **0.0009** | **0.069** |
| **Between place and object connectivity** | | **0.249** | 0.295 | **5.450** | **1,154** | **0.021** | **0.034** |

Table S6E: Adults – Comparison of the connectivity in each hemisphere in each network

Significant results are indicated in bold.

| Network connectivity | | Mean Right | Mean Left | Right-Left contrast | | | |
|---|---|---|---|---|---|---|---|
| | | | | F | DF | p | $\eta^2_p$ |
| Within face connectivity | 1. Core | **0.203** | 0.078 | **33.277** | **1,154** | **0.0000** | **0.178** |
| | 2. Extended | 0.121 | 0.083 | 2.808 | 1,154 | 0.0958 | 0.018 |
| | 3. Between core and extended | 0.047 | 0.028 | 2.268 | 1,154 | 0.134 | 0.014 |
| Within place connectivity | | 0.151 | 0.169 | 1.207 | 1,154 | 0.274 | 0.008 |
| Between core and place connectivity | | 0.075 | 0.051 | 3.692 | 1,154 | 0.056 | 0.023 |
| **Within object connectivity** | | **0.395** | 0.248 | **12.137** | **1,154** | **0.0006** | **0.073** |
| **Between core and object connectivity** | | **0.212** | 0.112 | **31.820** | **1,154** | **0.0000** | **0.171** |
| Between place and object connectivity | | 0.219 | 0.221 | 0.0125 | 1,154 | 0.911 | 0.000 |

**Control analyses**

To further validate our main results showing greater functional connectivity of the core face network in the right compared to the left hemispheres in all age groups, we conducted the following additional control analyses:

**1. Analysis of an independent dataset based on ABIDE2:**

To further validate our results on a different dataset of participants, we acquired data from the ABIDE2 [43]. Inclusion criteria was the same as in our main study (see details regarding the exclusion criteria below in *Methods- Preprocessing*). Note that the ABIDE2 database (similarly to the ABIDE) includes only data from children, adolescents, and adults.

Participants:

Table S7: General statistics of the participants from ABIDE2.

| **Age group** | **Number of participants** | **Age means** | **Age std.** |
|---|---|---|---|
| Adolescents | 10 | 14.206 | 1.982 |
| Adults | 22 | 25.225 | 5.807 |
| Children | 30 | 10.096 | 1.218 |

Results:

We first conducted a repeated measures ANOVA with the following between factors: group (children, adolescents, and adults, all from ABIDE2), within factors: network (as described in *Functional connectivity analysis*) and hemisphere. As in the original analysis reported in the paper, we did not find a main effect of group (F (2, 59) = 0.516, $p$=0.599, $\eta^2_p$ =0.017), but did find a main effect of network (F (7,413) = 84.323, $p$<0.000, $\eta^2_p$=0.588) and no interaction between these factors (F (14, 413) = 1.706, $p$=.052, $\eta^2_p$=0.055). We did not find a main effect of hemisphere (F (1, 59) = 2.228, $p$=0.141, $\eta^2_p$ =0.036), and no interaction between hemisphere and group (F (2, 59) = 3.027, $p$=0.056, $\eta^2_p$ =0.093). We found an interaction between network and hemisphere (F (7,413) = 7.624, $p$<0.000, $\eta^2_p$ =0.114). Finally, we did not find an interaction between network, hemisphere, and group (F (14, 413) = 0.8263, $p$=0.640, $\eta^2_p$ =0.028).

To examine the hemispheric dominance of each network in each age group, for each network in each age group we compared the right and the left functional connectivity (see more in *Tables S8A-C*). The connectivity of the core-face selective regions was larger in the right compared to the left hemisphere in all age groups. No right dominance was found within the place connectivity and within the object connectivity exhibited in all age groups (children, adolescents, and adults). The tables below also include analysis of hemispheric differences in the functional connectivity within the visual networks and between the visual network and within the extended face network (see *Tables S8A - C*).

Table S8A: Children – Comparison of the connectivity in each hemisphere in each network

Significant results are indicated in bold.

| Network connectivity | | Mean Right | Mean Left | Right-Left contrast | | | |
|---|---|---|---|---|---|---|---|
| | | | | F | DF | p | $\eta^2_p$ |
| Within face connectivity | 1. **Core** | **0.170** | 0.057 | **36.805** | **1,59** | **0.000** | **0.384** |
| | 2. Extended | 0.182 | 0.139 | 3.277 | 1,59 | 0.075 | 0.053 |
| | 3. Between core and extended | 0.080 | 0.075 | 0.140 | 1,59 | 0.709 | 0.002 |
| **Within place connectivity** | | 0.209 | **0.283** | **11.231** | **1,59** | **0.001** | **0.160** |
| Between core and place connectivity | | 0.115 | 0.094 | 1.683 | 1,59 | 0.200 | 0.028 |
| Within object connectivity | | 0.400 | 0.478 | 2.651 | 1,59 | 0.109 | 0.043 |
| **Between core and object connectivity** | | **0.267** | 0.214 | **5.196** | **1,59** | **0.026** | **0.081** |
| **Between place and object connectivity** | | 0.291 | **0.368** | **14.984** | **1,59** | **0.000** | **0.203** |

Table S8B: Adolescents – Comparison of the connectivity in each hemisphere in each network

Significant results are indicated in bold.

| Network connectivity | | Mean Right | Mean Left | Right-Left contrast | | | |
|---|---|---|---|---|---|---|---|
| | | | | F | DF | p | $\eta^2_p$ |
| Within face connectivity | 1. **Core** | **0.189** | 0.055 | **17.107** | **1,59** | **0.000** | **0.225** |
| | 2. Extended | 0.119 | 0.128 | 0.052 | 1,59 | 0.820 | 0.001 |
| | 3. Between core and extended | 0.082 | 0.042 | 2.353 | 1,59 | 0.130 | 0.038 |
| Within place connectivity | | 0.185 | 0.190 | 0.017 | 1,59 | 0.897 | 0.000 |
| **Between core and place connectivity** | | **0.101** | 0.034 | **5.760** | **1,59** | **0.020** | **0.089** |
| Within object connectivity | | 0.433 | 0.382 | 0.374 | 1,59 | 0.543 | 0.006 |
| Between core and object connectivity | | 0.285 | 0.219 | 2.664 | 1,59 | 0.108 | 0.043 |
| Between place and object connectivity | | 0.289 | 0.284 | 0.022 | 1,59 | 0.884 | 0.000 |

Table S8C: Adults – Comparison of the connectivity in each hemisphere in each network

Significant results are indicated in bold.

| Network connectivity | | Mean Right | Mean Left | Right-Left contrast | | | |
|---|---|---|---|---|---|---|---|
| | | | | F | DF | p | $\eta^2_p$ |
| Within face connectivity | 1. **Core** | **0.181** | 0.096 | **15.216** | **1,59** | **0.000** | **0.205** |
| | 2. Extended | 0.114 | 0.089 | 0.807 | 1,59 | 0.373 | 0.013 |
| | 3. Between core and extended | 0.015 | 0.011 | 0.065 | 1,59 | 0.800 | 0.001 |
| Within place connectivity | | 0.217 | **0.275** | **5.138** | **1,59** | **0.027** | **0.080** |
| **Between core and place connectivity** | | 0.111 | 0.105 | 0.113 | 1,59 | 0.738 | 0.002 |
| Within object connectivity | | 0.479 | 0.573 | 2.789 | 1,59 | 0.100 | 0.045 |
| Between core and object connectivity | | 0.259 | 0.257 | 0.005 | 1,59 | 0.943 | 0.000 |
| Between place and object connectivity | | 0.315 | 0.354 | 2.798 | 1,59 | 0.100 | 0.045 |

## 2. Time series signal-to-noise ratio (tSNR) analysis

To test whether greater functional connectivity in the right compared to the left core face network was not obtained due to differences in signal quality across the two hemispheres [46], we evaluated the time series signal-to-noise ratio (tSNR) across hemispheres in this network in the different age group. For each subject, we measured the tSNR during the resting state scans in each ROI of the core face network. As per previous publications, tSNR was computed for each voxel as follows: tSNR = mean(time series)/SD(time series) [45].

We first investigated the difference of the tSNR between the general tSNR in all the visual regions in the right hemisphere compared to the left hemisphere. Second, we investigated the tSNR within the core face regions in the right hemisphere compared to the left hemisphere. In both analyses we conducted a repeated measures ANOVA with the between factors: group (toddlers from Dinstein et al. [38], children from ABIDE, early adolescents from ABIDE, late adolescents from ABIDE and adults from ABIDE), within factors: hemisphere.

Within all the visual regions we did not find a main effect of group ($F(4,153) = 1.948$, $p=0.105$, $\eta^2_p=0.048$), neither a main effect of hemisphere ($F(1,153) = 1.671$, $p=0.198$, $\eta^2_p=0.011$) and no interaction between these factors ($F(4,153) = 1.721$, $p=0.148$, $\eta^2_p=0.043$). A comparison between the general tSNR of the right hemisphere and the left hemisphere in each group, only revealed that late adolescents had larger tSNR in the left hemisphere compared to the right hemisphere.

Next, we investigated the tSNR within the core-face regions. We did not find a main effect of group (F (4,153) = 1.875, $p$=0.118, $\eta^2_p$ =0.047), neither a main effect of hemisphere (F (1,153) = 0.033, $p$=0.855, $\eta^2_p$=0.000) and no interaction between these factors (F (4,153) = 0.871, $p$=0.483, $\eta^2_p$=0.022). A comparison between the general tSNR of the right hemisphere and the left hemisphere in each group revealed no difference between the tSNR of the right hemisphere compared to the left hemisphere in all age groups (see *Tables S9A & S9B*).

These results suggest that the right hemispheric dominance of the core-face regions that was found in the current study is not related to tSNR differences between the hemispheres.

Table S9A: Comparison of the tSNR within all visual region in each hemisphere in each age group

Significant results are indicated in bold.

| group | Mean Right | Mean Left | Right-Left contrast | | | |
|---|---|---|---|---|---|---|
| | | | F | DF | p | $\eta^2_p$ |
| Toddlers | 0.002 | 0.001 | 0.346 | 1, 153 | 0.557 | 0.002 |
| Children | 0.002 | 0.002 | 0.023 | 1, 153 | 0.880 | 0.000 |
| Early adolescents | -0.001 | -0.000 | 0.160 | 1, 153 | 0.690 | 0.001 |
| Late adolescents | 0.002 | **0.004** | **8.416** | **1, 153** | **0.004** | **0.052** |
| Adults | -0.000 | 0.000 | 0.542 | 1, 153 | 0.462 | 0.003 |

Table S9B: Comparison of the tSNR within all visual region in each hemisphere in each age group

Significant results are indicated in bold.

| group | Mean Right | Mean Left | Right-Left contrast | | | |
|---|---|---|---|---|---|---|
| | | | F | DF | p | $\eta^2_p$ |
| Toddlers | 0.002 | 0.002 | 0.012 | 1, 153 | 0.913 | 0.000 |
| Children | 0.002 | 0.001 | 1.580 | 1, 153 | 0.211 | 0.010 |
| Early adolescents | -0.001 | -0.001 | 0.002 | 1, 153 | 0.965 | 0.000 |
| Late adolescents | 0.001 | 0.002 | 1.672 | 1, 153 | 0.198 | 0.011 |
| Adults | -0.001 | -0.001 | 0.295 | 1, 153 | 0.588 | 0.002 |

**Assessing the contribution of different fROIs to the right lateralization of face functional connectivity:**

The main finding in this study is the greater functional connectivity of the core face network in the right compared to the left hemisphere. To deepen our understanding of the factors that contributed to this result,

we examined which edges between specific nodes of the core face network contributed the most to the right hemispheric lateralization. To do so, we used a linear discrimination analysis (LDA) [47]. The age of each participant and the functional connectivity between the fROIS of the visual selective regions (core-face, places and objects) were defined as classifiers of the right and left hemisphere. This model exhibited 80.818% classification accuracy, while testing the prediction with Leave One Out Cross-Validation (LOOCV) exhibited an accuracy level of 78.302%. *Table S10* describes which features donated most to this classification. Importantly, age had the lowest absolute value, suggesting that this feature shows the least contribution in predicting the hemispheric laterality. This further confirms our main result of right lateralization in all age groups. When examining the contribution of specific edges, we found that, within the core-face selective regions, the functional connectivity between the FFA and LOF, FFA and OFA, OFA and LOF, and LOF and pSTS had a positive and the highest absolute values. This suggests that these functional connections contributed the most to predicting the right hemispheric laterality. This result is consistent with the known role of these regions in face processing[10].

Table S10 - Coefficients of linear discriminants:

| visual category | Feature | LD1 | Hemisphere |
|---|---|---|---|
| core-face | FFA_LOF | 2.835 | R |
| core-face | FFA_OFA | 2.198 | R |
| core-face | OFA_LOF | 1.809 | R |
| core-face | LOF_pSTS | 1.308 | R |
| places | PPA_TOS | 0.919 | R |
| places | PPA_IPS | 0.794 | R |
| places | TOS_IPS | 0.342 | R |
| | age | 0.008 | R |
| objects | LOC_COS | -0.029 | L |
| places | PPA_ITS | -0.268 | L |
| places | ITS_TOS | -0.874 | L |
| places | ITS_IPS | -1.131 | L |
| core-face | OFA_pSTS | -1.513 | L |
| core-face | FFA_pSTS | -1.726 | L |
